# The COVID-19 Pandemic and Its Impact on Families’ Mental Health: The Role Played by Parenting Stress, Parents’ Past Trauma, and Resilience

**DOI:** 10.3390/ijerph182111450

**Published:** 2021-10-30

**Authors:** Eleonora Marzilli, Luca Cerniglia, Renata Tambelli, Elena Trombini, Leonardo De Pascalis, Alessandra Babore, Carmen Trumello, Silvia Cimino

**Affiliations:** 1 Department of Dynamic and Clinical Psychology, Sapienza University of Rome, 00186 Rome, Italy; eleonora.marzilli@uniroma1.it (E.M.); renata.tambelli@uniroma1.it (R.T.); 2 Faculty of Psychology, International Telematic University Uninettuno, 00186 Rome, Italy; l.cerniglia@uninettunouniversity.net; 3 Department of Psychology, University of Bologna, 40127 Bologna, Italy; elena.trombini@unibo.it (E.T.); leonardo.depascalis@unibo.it (L.D.P.); 4 Laboratory of Dynamic Psychology, Department of Psychological Sciences, Health and Territory, Università degli Studi “G. d’Annunzio” Chieti-Pescara, 66100 Chieti, Italy; alessandra.babore@unich.it (A.B.); carmen.trumello@unich.it (C.T.)

**Keywords:** COVID-19, peritraumatic distress, parenting stress, trauma, resilience

## Abstract

International research has evidenced the psychological impact of the COVID-19 pandemic on families, and the key role played by parenting stress levels. Although significant associations with parents’ past trauma and resilience have been shown, this study aimed to explore their complex interplay on the relationship between parents’ peritraumatic distress due to COVID-19, parenting stress, and children’s psychopathological difficulties. We recruited 353 parents with children aged two to 16 years via an online survey during the Italian second wave of COVID-19. Parents’ peritraumatic distress due to COVID-19, parenting stress, past trauma and resilience, and children’s psychological difficulties were assessed through self-report and report-form questionnaires. Parents’ past traumas significantly predicted peritraumatic distress due to COVID-19 and children’s psychological difficulties. The relationship between past traumas and children’s psychological difficulties was serial mediated by parents’ peritraumatic distress and parenting stress. Direct and total effects of parent’s resilience on parent’s peritraumatic distress were not significant, but there were significant indirect effects via parenting stress and via parents’ peritraumatic distress and parenting stress, indicating inconsistent mediation. This study evidenced the key risk and protective role played by, respectively, parents’ past traumas exposure and resilience on the relationship between parents’ psychological difficulties due to COVID-19, parenting stress, and children’s psychological difficulties, with important clinical implications.

## 1. Introduction

### 1.1. The Impact of the COVID-19 Pandemic on Families’ Daily Life

In December 2019, the CoronaVirus Disease 19 (COVID-19) appeared in Wuhan, China. Due to its rapid diffusion, on 11 March 2020, it was declared as a global pandemic by the World Health Organization (WHO). To prevent its spread, the Governments of many countries worldwide, including Italy, planned a series of containment measures (e.g., physical distancing, school closures, and work from home), which severely affected the habits of individuals’ everyday lives, especially families with pre-school- and school-aged children [1,2,3]. Indeed, in addition to the resulting limitations of freedom, and health and economic concerns, parents of children aged from early childhood to middle adolescence [4,5,6] had to face a dramatic increase in the management of daily family life, their children’s care, and associated stress levels [7]. Due to the home confinement, the closure of educational services, and the lack of children’s interactions with teachers and peers, parents found themselves alone in the promotion of new learning experiences for their children [3], and in the management of home-schooling and social activities for school-aged children [4]. Moreover, parents with early and middle adolescents (approximately from 11 to 16 years) [8] have had to face a substantial increase of conflicts with their children and, consequently, of their stress levels [6,9]. This could be due to the increment in the amount of time spent together during a stage of life in which, physiologically, adolescents tend to become more independent from parents, preferring to socialize with peers [10,11]. Overall, as suggested by Coyne et al. [12], parents have experienced a stressful condition of collision of roles and responsibilities, having to take care of their children’s education and, at the same time, of work, marital and domestic burdens, with important consequences of their psychological well-being.

### 1.2. Psychopathological Symptoms Due to COVID-19 in Parents and Children, and Parenting Stress

The restrictive measures put in place to stem the diffusion of the COVID-19 virus have implied an important impact on families’ everyday functioning [1,2,3]. Nevertheless, some families have shown adaptive responses to the adverse circumstances imposed by the COVID-19 preventive strategies [13], maintaining a positive involvement with family members and the extra-family social network [14,15], and experiencing a sense of self-efficacy [2]. Nevertheless, national and international research reported that a large part of families of the general population experienced the pandemic as a traumatic event, manifesting anxiety, depression [16], and peritraumatic distress symptoms [17,18]. In this context, the few studies conducted during the so-called “second wave” of COVID-19 have confirmed the long-term effects on mental health and increased psychopathological symptoms as the pandemic continues [18,19,20]. Moreover, studies have evidenced the presence of psychological suffering due to the COVID-19 outbreak among children as well [21,22], with higher emotional and behavioral symptoms, and social problems with peers [23,24,25]. Beyond the direct effects of COVID-19 on children’s psychological well-being, a key role of parents’ perception of the pandemic as a traumatic event and the resulting peritraumatic distress has also been reported [17]. In this context, clinicians and researchers in the field of developmental psychopathology have shown that children tend to react to a stressful event (such as the COVID-19 pandemic) based on their parents’ interpretation and emotional responses [26]. Specifically, it has been evidenced the presence of intergenerational transmission of psychological symptoms from parents to children [27], also in the context of the psychological responses to the COVID-19 pandemic [5,18,28,29]. Parents who experience COVID-19 as a traumatic experience, manifesting peritraumatic symptoms in response to the pandemic and its restrictions, may transmit the same maladaptive emotional-behavioral responses to their children [17]. Consequently, given the clinical relevance of the phenomenon, it is important to implement the knowledge of underpinning mechanisms that may promote or mitigate the short- and long-term consequences of the COVID-19 pandemic on families’ psychological well-being, to promote the planning of effective and more targeted interventions.

One of the main mechanisms through which parents’ psychopathological symptoms may affect children’s emotional-behavioral functioning is by the negative affective environment provided to their children, especially in terms of a poor quality of parent-child relationship and higher levels of parenting stress [30,31]. In the specific context of the COVID-19 pandemic, many studies have evidenced a significant increase in parenting stress levels in parents [5,18,20,29], which in turn represent crucial risk factors for children’s psychopathological difficulties. In accordance with spillover models [32,33], stress that a parent arises in response to stressful contextual factors (e.g., pandemic-related negative circumstances) may also trigger negative affective responses when parents interact with their children, leading to increased parenting stress levels. International research has widely shown that parenting stress represents a crucial risk factor for children’s psychopathological difficulties [31], and that the influence of parental psychopathological symptoms on children’s psychopathological symptoms could be mediated by parenting stress levels [34]. Recently, in the specific context of the COVID-19 pandemic, many studies have also reported a worrying increase in parenting stress levels [20], suggesting that pandemic-related negative life events may also have indirect effects on children. In line with this, recent studies have confirmed the significant mediation role played by parenting stress on the relationship between parents’ psychopathological symptoms resulting from COVID-19 and children’s psychopathological difficulties [5,18,29].

### 1.3. The Role of Parents’Past Trauma and Resilience, and Their Complex Interplay with Parents’ Psychopathological Symptoms Due to COVID-19 and Parenting Stress

International literature focused on the psychological impact of the COVID-19 pandemic on families has reported psychopathological symptoms in parents and children [16,17,18,19,20,21,22,23,24,25], and that parents’ psychopathological problems due to COVID-19 may affect children’s psychological functioning both directly than via high parenting stress levels [5,18,28,29]. To prevent the short- and long-term negative outcomes related to these processes on parents’ and children’s psychological well-being, it is important to increase the knowledge on families’ factors that may exacerbate or mitigate the risk exerted by psychopathological risk and parenting stress experienced by parents during the pandemic.

According to the Developmental Psychopathology framework [35], psychopathological difficulties in parents and children are the result of the dynamic interplay between individual and relational protective and risk factors, from individual vulnerabilities to parents’ strengths. Among individual vulnerabilities, some studies have shown that having experienced previous trauma may lead the individual to a greater vulnerability to the effects of subsequent traumatic experiences, with a higher risk of developing post-traumatic stress symptoms [36,37,38]. The stress sensitization model posited that an individual who has higher levels of prior trauma exposure might manifest higher sensitivity and lower tolerance to stress when exposed to later stressful life events [33,39]. Recently, the same associations were also found in relation to the COVID-19 pandemic. Specifically, the studies by Lahav [40] and by John-Henderson and Ginty [41] have reported higher peritraumatic distress symptoms resulting from COVID-19 among individuals with a history of trauma exposure, suggesting that parent’ past trauma may represent a significant risk factor for emotional and behavioral response to COVID-19 pandemic. Interestingly, scientific literature has also shown that parental history of trauma exposure is prospectively associated both with higher levels of parenting stress [42], and with children’s psychopathological problems [43], and that the relationship between these variables is not linear, but complex and dynamic [44]. However, to date no study has yet explored the possible influence exerted by parents’ past trauma on children’s psychological problems during the pandemic, considering its complex interplay with parents’ peritraumatic distress due to COVID-19 and the resulting increase in parenting stress levels. As evidenced above, parents’ psychopathological symptoms due to COVID-19 may lead to children’s psychopathological symptoms both directly than through parenting stress levels [5,18,28,29]. The findings of previous studies seem to suggest that parents’ past trauma may further exacerbate these risk influences on parents’ and children’s well-being, supporting the need to further explore these relationships.

On the other hand, parents’ resilience has been suggested to be a crucial protective factor for the psychological adjustment to COVID-19 [6,45]. Specifically, resilience refers to the individual’s ability to successfully cope with stressful experiences [46], and many studies have underlined its key role in adapting to other pandemics and disasters [47,48]. From a family point of view, parents’ resilience involves the family’s capacity to make a sense of an adverse experience and to adapt to the stressor [49]. Recently, the same protective role was also found in response to the pandemic [50], with lower psychopathological symptoms due to COVID-19 in parents with higher resilience abilities [6,45]. Moreover, high levels of parental resilience have been associated with lower levels of parenting stress [51] and lower children’s psychopathological difficulties [52]. However, despite this promising evidence, no study has yet explored the possible protective role played by parents’ resilience on the complex interplay between parents’ psychological symptoms due to COVID-19, parenting stress, and children’s psychopathological difficulties [5,53].

### 1.4. The Current Study

During the COVID-19 pandemic, a growing body of research has shown increased psychopathological symptoms among parents and children [16,17,18,19,20,21,22,23,24,25], and significant associations with parenting stress levels [5,18,20], parents’ past trauma [40,41], and parents’ resilience [6,45,50]. The significant mediation role played parenting stress levels on the relationship between parent’s psychopathological symptoms due to COVID-19 pandemic and children’s psychopathological problems has also been established [5,18,28,29]. Interestingly, recent literature has shown that parents’ prior trauma significantly predicted higher psychopathological symptoms due to COVID-19 in parents [40,41], higher parenting stress levels [42], and children’s psychopathological difficulties [43], whereas parents’ resilience has been shown to be negatively associated with the same outcomes [45,51,52]. This evidence suggested a possible, but as yet unexplored, serial mediation of parents’ psychological impact of COVID-19 and parenting stress on the risk and protective role played by, respectively, parental history of trauma and resilience on children’s psychopathological symptoms.

Based on the previous theoretical and empirical premises, this study aimed to explore whether parents’ peritraumatic distress due to COVID-19 and parenting stress simply and serially mediated the effects of parents’ past traumas exposure and parental resilience on children’s psychopathological difficulties. Specifically, we hypothesized that: (a) Parents’ resilience may mitigate the risk effects exerted by the psychopathological impact of COVID-19 in parents and parenting stress, whereas; (b) higher parents’ past traumas may represent an additional risk factor in these processes.

## 2. Materials and Methods

### 2.1. Participants

From 10 October 2020 to 15 January 2021, during the Italian second wave of COVID-10, we recruited *n* = 487 parents of children aged 2–16 years via social media (i.e., Facebook) and via notices posted on online psychology research websites. We conducted an online convenience sampling to collect the data. All parents who decide to participate in the study signed a written informed consent in which the steps of the study were explained. The study was approved by the Ethical Committee of the Department of Dynamic and Clinical Psychology at Sapienza University of Rome (protocol N. 809/2020), in accordance with the Declaration of Helsinki.

The inclusion criteria of the sample were: The age range of children from 2 to 16 years; the absence of physical or mental disorders in parents and/or children; the absence of children and/or parents who were following a psychiatric or psychological treatment. From the total sample, we excluded the following cases: *n* = 23 parents with mental and/or physical disability, and *n* = 19 parents of children with psychiatric and/or physical diagnoses; *n* = 21 parents and *n* = 18 parents of children who were undergoing psychological and/or psychiatric treatment. Finally, *n* = 53 parents who did not complete the assessment procedure were also excluded. The final sample included of *n* = 353 parents (64% mothers; Mage = 42.15, SD = 8.09) with children aged between 2 and 16 years (Mage = 9.28, SD = 4.54; 50.7% females). All parents lived in Italy and most of them were married (70.5%). The majority had high school (43.6%) or more than high school (50.7%) level of education, and most often (43.6%) reported a household income between 55,000 and 75,000 euros per year.

### 2.2. Procedure

After giving their written consent to participate in the study, parents filled out an online survey composed of an ad-hoc questionnaire assessing sociodemographic information (See Supplementary Material S1 for more details), followed by the COVID-19 *Risk Index* questionnaire, an ad-hoc questionnaire for the evaluation of possible changes in families’ everyday lives due to the COVID-19 outbreak (See Supplementary Material S2 for more details). Then, parents filled out self-reported and report-form instruments (described below) assessing their psychological distress due to COVID-19, previous traumatic experiences, resilience, parenting stress, and children’s psychopathological difficulties.

### 2.3. Measures

#### Questionnaire Design

The online questionnaire was designed to be filled out by parents of children between 2 and 16 years old of the general population. It is composed of four sections.

The first section collected sociodemographic data (e.g., sex, age, educational background, marital status, child’s age and sex, etc.) (See Supplementary Material S1 for more details).

The second section included questions specifically related to possible changes in daily life resulting from the COVID-19 pandemic and the psychological impact of COVID-19 on parents. Specifically, first parents filled out the COVID-19 Risk Index questionnaire, an ad-hoc self-report instrument questionnaire that we computed on the basis of previous studies in this field [2,5,18,29] to evaluate possible changes in families’ everyday lives due to the COVID-19 outbreak. It is composed of 12 items assessing: possible changes in parents’ work due to COVID-19 (i.e., no changes; smart working; loss of work); changes in their children instruction and education (i.e., distance learning); having to take care of their children’s education, for many hours; whether there was someone to help them in the domestic management of their children; whether they, a familiar, or a close friend resulted positive from COVID-19 infection, and/or died as a consequence of COVID-19 (See Supplementary Material S2 for more details). Then, parents filled out the COVID-19 Peritraumatic Distress Index (CPDI) [54,55], a self-report questionnaire for the evaluation of peritraumatic distress symptoms due to COVID-19. Specifically, items assessed symptoms of anxiety, depression, specific phobia, avoidance and/or compulsive behaviors, in accordance with criterion A for Post-Traumatic Stress Disorder (PTSD). A total score ranging 0–100 was created by summation. Higher scores are indicative of higher levels of psychological distress. The CPDI showed good internal coherence both in previous studies [54,55], and in the present one (Cronbach alpha = 0.87).

The third section assessed parents’ individual variables that we considered as possible risk and protective factors for parents’ and children’s psychological adaptation to the COVID-19 pandemic (i.e., the presence stressful and/or traumatic experiences in the parents’ past, and parents’ resilience abilities). Specifically, the Traumatic Experiences Checklist (TEC) [56] is a retrospective self-report measure investigating 29 types of potential trauma and overwhelming experiences. Different scores can be calculated on the TEC. For the aims of this study, we used the total trauma cumulative score. The TEC [56] and the Italian translation [57] have been shown to have good psychometric properties. In the current study, the Cronbach’s alpha was also adequate (Cronbach alpha = 0.85). The Connor–Davidson Resilience Scale (CD-RISC) [58] is a self-report questionnaire assessing the ability to cope with adversity among general and clinical populations. It is composed of 25 items, evaluated on a scale from 0 = “not true at all” to 4 = “true nearly all of the time”. The scores were summed to compute the total score, with higher scores indicating greater resilience. The CD-RISC has been shown to have good internal consistency and test–retest reliability, both previously [58] and in this study (Cronbach alpha = 0.83).

Finally, in the fourth section, parents are asked to report on the stress they perceive in their relationship with their child and on child’s emotional-behavioral functioning. Specifically, the Parenting Stress Index-Short Form (PSI-SF) [59,60] is a self-report questionnaire, composed of 36 items, for the assessment of parental stress. The scores were summed to calculate Total scores, with higher scores indicating higher parenting stress levels. In this study, the internal consistency of the scale was good (Cronbach alpha = 0.85). The Strengths and Difficulties Questionnaire (SDQ) [61,62] is a self-report questionnaire for the assessment of children’s psychological difficulties. It is composed of five subscales (conduct problems, hyperactivity, emotional symptoms, peer problems, and prosocial behavior). Summing the scores of all problems scales, it is possible to obtain a total score indicative of the child’s psychopathological difficulties, which we used in this study. The scale showed very good internal consistency, with a Cronbach alpha = 0.81 in this study.

### 2.4. Statistical Analyses

All analyses were performed using SPSS software, Version 26 (IBM, Armonk, NY, USA), Preliminary statistical analyses were conducted using descriptive statistics (reliability of the measures, frequencies, mean scores, and percentages). To compute a COVID-19 Risk Index score from our ad-hoc questionnaire, a score (0, 0.5, 1, or 2 points) was assigned to each item based on the degree to which the related change due to COVID-19 affected parents’ psychological-adaptive functioning. The score for each item was summed to obtain a total COVID-19 Risk score. Then, to determine initial significant correlations between study variables, and relevant covariates, Pearson’s correlation analyses were carried out. Based on significant correlations that we found, hierarchical multiple regression analyses were carried out to identify the main effects of parents’ peritraumatic distress due to COVID-19, past trauma, and parenting stress on children’s psychological difficulties, controlling for relevant covariates. Finally, to verify whether parents’ peritraumatic distress due to COVID-19 and parenting stress sequentially mediated the relationship between parents’ past trauma and resilience with children’s psychopathological difficulties, two sequential mediation analyses were performed. Mediation analyses were conducted using Hayes’s [63] PROCESS macro (Model 6), evaluating indirect effects with 95% bias-corrected confidence intervals (CIs) based on 10,000 bootstrap samples.

## 3. Results

### 3.1. Association between the Variables under Study

Results showed that parents’ peritraumatic distress due to COVID-19 was significantly associated with parents’ traumatic experiences, parents’ resilience, parenting stress, and children’s adaptive difficulties. Moreover, both parents’ scores on CPDI and PSI were significantly associated with each other, and both with children’s SDQ total score, supporting the possibility of their sequential mediating role in the relationships between parents’ traumatic experiences and resilience with children’s adaptive difficulties (Table 1).

### 3.2. Main Effects of Parents’ Peritraumatic Distress Due to COVID-19, Past Trauma, and Parenting Stress on Children’s Psychological Difficulties

Based on significant correlations that emerged, hierarchical multiple regression analyses were conducted to verify whether parents’ peritraumatic distress due to COVID-19, past trauma, and parenting stress levels were predictive of children’s psychological difficulties. Given that parents’ COVID-19 Risk Index, parents’ sex, and children’s age showed significant associations with the score of SDQ in previous Pearson’s correlation analyses, regression analyses were adjusted for these covariates. As possible to see in Table 2, high levels of past traumas and parenting stress were significantly predictive of high children’s psychological difficulties. Conversely, the relationship between parents’ peritraumatic distress due to COVID-19 and children’s psychological difficulties was not significant. Finally, the child’s age was confirmed to be a significant covariate and was inserted as a covariate in mediation analyses. The model accounted 10% of the variance.

### 3.3. Parents’ Peritraumatic Distress Due to COVID-19 and Parenting Stress as Serial Mediators for Effects of Parents’ Traumatic Experiences and Resilience on Children’s Psychopathological Difficulties

**Figure 1:**
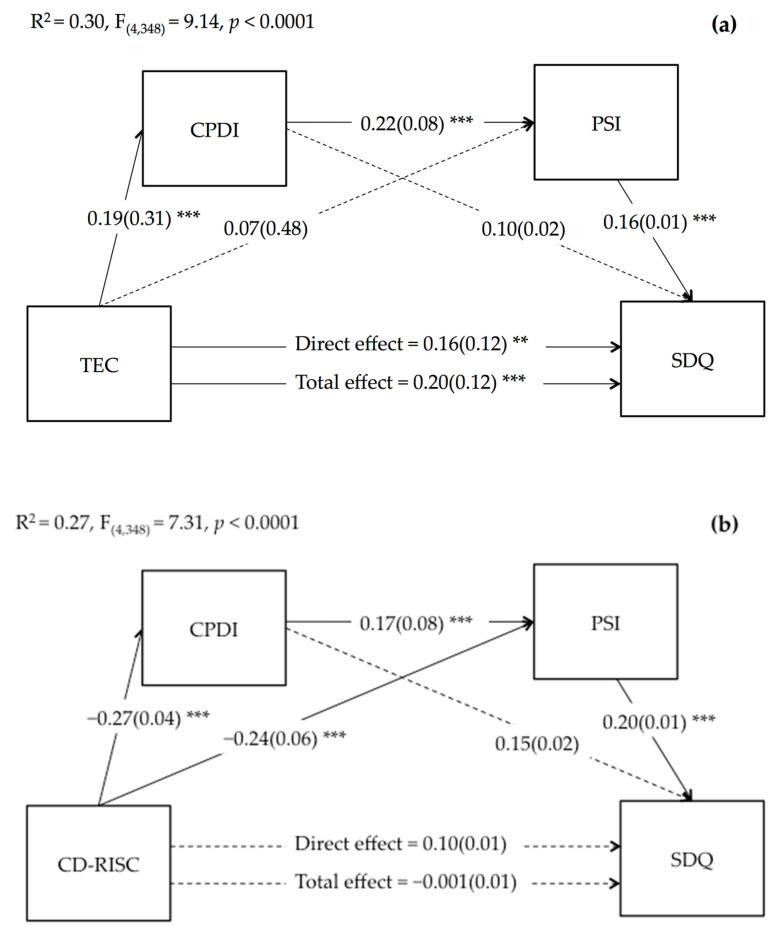
Serial mediation of parent’s COVID-19 peritraumatic distress (CPDI) and parenting stress (PSI) on the relationship between parent’s traumatic experiences (TEC) (**a**) and parent’s resilience (CD-RISC) (**b**) with children’s difficulties (SDQ). Coefficients shown are standardized path coefficients. Dotted lines represent non-significant parameters. ** *p* < 0.01, *** *p* < 0.001.

Finally, we verified whether parents’ peritraumatic distress due to COVID-19 and parenting stress simply and serially mediated the effect of parent’s traumatic experiences and resilience on children’s difficulties. Regarding parents’ traumatic experiences, results presented in Figure 1a show that both the direct and total effects of TEC on children’s difficulties were significant. However, considering the effects of mediators, the effect of the direct effect was smaller than the total effect. Moreover, TEC significantly predicted CPDI, but not PSI. The direct effect of PSI on children’s difficulties was also significant, whereas the direct effect of CPDI was not. In addition, parents’ CPDI, as the first mediator, significantly predicted PSI, as the second mediator.

As shown in Table 3, the indirect paths via multiple serial mediation of parents’ peritraumatic distress due to COVID-19 and parenting stress were statistically significant, explaining 4% of the total effect. Conversely, the single mediations of the two mediators were not significant.

Regarding parents’ resilience, as shown in Figure 1b, parents’ resilience significantly predicted low levels of parent’s peritraumatic distress due to COVID-19 and parenting stress. Moreover, high levels of parents’ peritraumatic distress due to COVID-19 and parenting stress significantly predicted high children’s difficulties, whereas both the direct and total effects of parents’ resilience on children’s difficulties were not significant. In addition, as shown in Table 2, the indirect paths via simple mediations of parents’ peritraumatic distress and parenting stress and via multiple serial mediation of parents’ peritraumatic distress due to COVID-19 and parenting stress were statistically significant, indicating inconsistent mediations. The simple and multiple serial mediations explained, respectively, 35% and 10% of the total effect.

## 4. Discussion

The present study aimed to further enhance knowledge on possible risk and protective factors implied in the psychopathological impact of COVID-19 among families with children. Research has shown that parents with developing children (especially from early childhood to middle adolescence) represent the population group most at risk from the psychological effects of COVID-19, also due to the increased responsibility associated with their children’s developmental phase-specific needs, on which COVID-19-restrictions have had a profound impact [3,4,5,6,29]. Specifically, we chose to explore the role played by parenting stress, parents’ past trauma exposure, and resilience, based on previous literature showing their key contribution in the transmission of psychopathological risk from parents to children [42,43,52,53], including the psychopathological impact of the COVID-19 pandemic [40,41,50]. Although previous studies have suggested that the relationship between parents’ and children’s psychopathological difficulties due to COVID-19 may be mediated by parenting stress levels [5,18,29], to the best of our knowledge, the present study is the first study to explore the possible risk and protective role played by, respectively, parents’ past trauma exposure and resilience in these processes.

Overall, our findings confirmed what was expected. In particular, results of our mediation analyses, after controlling for confounding covariates, showed that higher parents’ peritraumatic distress resulting from COVID-19 significantly predicted higher levels of parenting stress, that in turn are significantly, and positively, associated with higher children’s psychopathological difficulties. These findings are in accordance with the studies by Chartier and coll. [17] and by Czeisler and coll. [16] that have shown a deterioration in parents’ psychological well-being due to COVID-19 pandemic, which significantly affected children’s psychological well-being through the resulting increase of parenting stress levels [5,29,53]. In this field, clinicians and researchers rooted in the Developmental Psychopathological framework have widely suggested that parental psychopathological difficulties may affect children’s emotional-adaptive functioning in a cascading way [64], from the transmission of predisposition to (epi-)genetic vulnerabilities [65,66], to children’s exposure to an adverse affective family environment [67,68,69], including poor quality of parent-child interactions [30,70,71], family functioning [72,73,74,75], and high parenting stress levels [76,77]. Recently, cascading effects have also been suggested in the context of COVID-19: The psychological impact of COVID-19 on parents may negatively influence parenting and related stress levels, which in turn may place children at higher risk of psychopathological symptoms [50].

Beyond the complex relationship yet established between parents’ psychopathological symptoms due to COVID-19, parenting stress levels, and children’ psychological difficulties [5,18,28,29], that also this study has confirmed, our findings added, to previous literature, new evidence on the crucial role played by parents’ past trauma and resilience in exacerbating/mitigating these cascading effects. In particular, regarding parents’ previous traumas, we found that parents’ prior trauma exposure significantly predicted children’s psychological difficulties both directly, and via the serial mediation of parents’ peritraumatic distress due to COVID-19 and parenting stress. The direct effect of parents’ past traumas on children’s psychological difficulties is in line with previous studies [43]. However, our results have increased the knowledge on the contribution played by parents’ history of trauma on parents’ psychopathological symptoms due to COVID-19 and parenting stress, as well its complex relationship with these yet established risk factors in shaping children’s psychological difficulties. Indeed, parents’ past trauma significantly predicted parents’ peritraumatic distress due to COVID-19, but the relationship with parenting stress was not significant. International research has also shown that having experienced previous trauma may lead the individual to a greater vulnerability to the effects of subsequent traumatic experiences [33,39]. In this field, neuroscientific studies have suggested that prior trauma exposure may sensitize the autonomic nervous system, leading to altered mental and physical responses in the face of a future additional stressor [78]. However, only a few studies have focused on the role played by previous trauma when facing implications of the COVID-19 pandemic in facing the COVID-19 pandemic [40,41], and our is the first study to explore these processes among a sample of parents. On the complex interplay between these variables, we found that the effect of parents’ prior trauma on children’s psychological well-being was reduced when considering the role played by parents’ peritraumatic distress and parenting stress, suggesting a partial mediation role of these variables. In addition, although parents’ past traumas did not directly predict parenting stress levels, this study is the first to evidence its indirect effect through the risk influence exerted on parents’ peritraumatic distress due to COVID-19 (as the first mediator), that in turn predicted parenting stress levels (as the second mediator). In line with our hypothesis, our findings confirmed that parents’ previous traumas exposure represents an additional risk factor for the negative influence exerted by parents’ psychopathological symptoms due to COVID-19 and parenting stress on children’s psychological functioning, providing new knowledge on risk factors involved in the psychological well-being of families during the pandemic.

On the other hand, past literature following other disasters and pandemics has demonstrated the key role played by resilience in mitigating the negative short- and long-term effects of stress on parents and children [47,48]. Our results further point in this direction, in relation to the specific context of the COVID-19 pandemic as well. Specifically, we found that higher levels of resilience in parents were predictive of lower levels of parents’ peritraumatic distress due to COVID-19 and parenting stress. Interestingly, parents’ resilience was not directly associated with children’s psychopathological difficulties, whereas the indirect effects via peritraumatic distress due to COVID-19 and via parenting stress and multiple serial mediation of parents’ peritraumatic distress and parenting stress were statistically significant, indicating inconsistent mediation [79]. These findings are in line with the studies by Lahav [40] and by John-Henderson and Ginty [41] that have shown that higher levels of resilience are associated with lower individuals’ psychopathological symptoms resulting from COVID-19, including anxiety/depressive symptoms, and PTSD symptoms. Moreover, the protective role of parents’ resilience on parenting stress levels [51] and children’s psychological well-being [52] was also shown. Notably, as evidenced above, international literature focused on the psychological impact of the COVID-19 pandemic on families has shown that parents’ psychopathological symptoms due to COVID-19 may lead to children’s psychopathological symptoms directly rather than through parenting stress levels [5,18,29], supporting the importance of implementing the research on possible factors that may mitigate this worrying negative developmental cascade. In line with this, ours is the first study to evidence the key protective role played by parents’ resilience in these processes. Specifically, our findings suggested that parents’ resilience may mitigate the risk exerted by parents’ peritraumatic distress due to COVID-19 and parenting stress (as well as their prospective association) on children’s psychological problems, with important clinical implications.

This study had a number of limitations. We used self-report and report-form instruments for the assessment of the variables under study. These instruments are extensively validated and used, but further research should confirm our findings using more robust measures (e.g., clinical interviews). Moreover, there was no pre-COVID-19 assessment, which warrants caution in interpreting the reported effects as being consequences of the COVID-19 pandemic. In addition, given the cross-sectional design of this study, further studies using longitudinal designs are needed to confirm the hypothesized causal link between variables and the cascading manner in which they exert their effects. Notwithstanding the above limitations, this was the first study to explore the complex relationship between parents’ peritraumatic distress due to COVID-19, parenting stress, and children’s psychological problems, considering the possible risk and protective role exerted, respectively, by parents’ past traumatic experiences and resilience. Our findings have evidenced a key role of parent’s past traumatic experiences in exacerbating the negative effect exerted by parents’ peritraumatic distress due to COVID-19 and parenting stress on children’s psychopathological difficulties. On the other hand, parental resilience was found to exert a mitigation effect on the risk influences played by parental psychopathological difficulties due to COVID-19 and parenting stress on children’s psychological well-being.

## 5. Conclusions

Recent international research on the psychopathological impact of the COVID-19 pandemic and its related restriction on families has evidenced the significant contribution played by parents’ past trauma, resilience, and parenting stress. However, this is the first study to explore the complex interplay between these variables, evidencing a significant risk and protective role exerted by, respectively, parents’ past trauma and resilience. These findings could inform the planning of treatments and preventive programs, improving their effectiveness in promoting families’ psychological well-being in the face of COVID-19. Specifically, our results add new evidence on the importance of interventions focused on parenting support to reduce the long-term effect of the COVID-19 crisis on families and the transmission of intergenerational trauma. In fact, given that parental stress emerged as a crucial negative sequela of the pandemic for both parents and children, family-based intervention aimed at promoting parents’ intra- and inter-personal resources are called for [50,80,81]. To this end, before the COVID-19 pandemic, family-based preventive and intervention programs provided by telehealth services have shown their effectiveness in promoting positive parenting behaviors and supporting parents’ and children’s psychological well-being [82,83]. Consequently, these should be also promoted in time of COVID-19. Moreover, our findings have evidenced that parents with histories of trauma, and their children, are at higher risk for stress and psychopathological symptoms resulted from the pandemic. Consequently, intervention programs specifically targeted at caregivers with traumatic history are called for to promote positive family adaptation to the pandemic and reduce the short- and long-term consequences on parents and children’s psychological well-being. At the same time, this study offers further evidence that parents’ resilience plays a key role in how families cope with the COVID-19 outbreak. During emergencies, such as the pandemic, parents’ resilience has shown to have a positive effect on both parents and children, with positive mental health outcomes. Intervention programs specifically aimed at promoting feelings of resilience have been shown to be effective in reducing psychopathological problems during previous pandemics [84]. Consequently, preventive programs should be focused on the improvement of resilience and reinforcement of parents’ resources that may provide a protective effect on the family environment to promote the mental health of their members.

## Figures and Tables

**Table 1:** Pearson correlation coefficients between the starting theoretical model variables.

	1.	2.	3.	4.	5.	6.	7.	8.	9.	10.
1. Parent’s sex	1									
2. Parent’s age	0.15 **	1								
3. Child’s age	0.15 **	0.52 **	1							
4. Child’s sex	0.07	−0.01	−0.06	1						
5. COVID-Risk Index	−0.06	0.06	0.17 **	−0.003	1					
6. CPDI	−0.25 **	−0.01	−0.002	−0.09	0.19 **	1				
7. TEC	−0.06	−0.02	−0.11 *	0.01	0.06	0.19 **	1			
8. CD-RISC	0.05	0.02	0.05	−0.02	0.003	−0.27 **	−0.07	1		
9. PSI	0.04	0.02	0.15 **	−0.01	0.04	0.23 **	0.10	−0.28 **	1	
10. SDQ	−0.14 **	−0.007	−0.09	−0.006	0.07	0.17 **	0.21 **	−0.007	0.19 **	1

Note. CPDI = COVID-19 Peritraumatic Distress Index; TEC = Traumatic Experiences Checklist; CD-RISC = Connor–Davidson Resilience Scale; PSI = Parenting Stress Index-Short Form; SDQ = Strengths and Difficulties Questionnaire; * *p* < 0.05, ** *p* < 0.01.

**Table 2:** Results of hierarchical multiple regression analyses predicting children’s psychological difficulties.

		Adjusted Coefficients		
		*B*	*t*	*p*
Covariates				
	Parents’ age	0.06	1.05	0.29
	Children’s age	−0.14	−2.26	0.02 *
	COVID-Risk Index	0.06	1.16	0.24
Predictors				
	TEC	0.16	3.05	0.002 **
	CPDI	0.09	1.72	0.08
	PSI	0.17	3.24	0.001 ***
	R^2^			0.10
	R^2^ change			0.08

Note. TEC = Traumatic Experiences Checklist; CPDI = COVID-19 Peritraumatic Distress Index; PSI = Parenting Stress Index-Short Form; Coefficients shown are standardized regression coefficients. * *p* < 0.05, ** *p* < 0.01, *** *p* < 0.001.

**Table 3:** Indirect effects of parents’ traumatic experiences and resilience on children’s difficulties through parent’s peritraumatic distress due to COVID-19 and parenting stress.

Indirect Effect	Effect (BootSE)	LLCI	ULCI
TEC › CPDI › SDQ	0.04(0.03)	−0.003	0.12
TEC › PSI › SDQ	0.03(0.02)	−0.01	0.10
TEC › CPDI › PSI › SDQ	0.01(0.01)	**0.01**	**0.04**
CD-RISC › CPDI › SDQ	−0.01(0.01)	**−0.03**	**−0.002**
CD-RISC › PSI › SDQ	−0.01(0.01)	**−0.03**	**−0.003**
CD-RISC › CPDI › PSI › SDQ	−0.01(0.01)	**−0.01**	**−0.001**

Note. TEC = Traumatic Experiences Checklist; CPDI = COVID-19 Peritraumatic Distress Index; PSI = Parenting Stress Index-Short Form; SDQ = Strengths and Difficulties Questionnaire; CD-RISC = Connor–Davidson Resilience Scale; BootSE = Boot-strapped standard error; LLCI = Lower level confidence interval; ULCI = Upper level confidence interval. All bold values are statistically significant.

## Data Availability

The data presented in this study are openly available in FigShare at doi:10.6084/m9.figshare.16617682.

## Supplementary Materials

The following are available online at www.mdpi.com/article/10.3390/ijerph182111450/s1, Supplementary Material S1. Socio-demographic questionnaire; Supplementary Material S2. Covid-19 Risk Index questionnaire.
