# Supplementary material for: The COVID-19 Pandemic and Its Impact on Families’ Mental Health: The Role Played by Parenting Stress, Parents’ Past Trauma, and Resilience
Source: Int J Environ Res Public Health. 2021 Oct 30;18(21):11450. doi: 10.3390/ijerph182111450 (PMC8583183; doi:10.3390/ijerph182111450)
Supplement: Supplementary file 1 [file ijerph-18-11450-s001.zip › ijerph-1401373-supplementary.pdf]

Supplementary Materials

Supplementary Material 1. Socio-demographic questionnaire

1.	Sex	A. Female<br>B. Male
2.	Age	____________
3.	Relationship status	A. Married<br>B. Cohabit<br>C. Separated/Divorced<br>D. Single<br>E. Other:________
4.	Educational levels	A. Less than high school<br>B. High school<br>C. More/post more than high school
5.	Family Income	A. 0–15,000<br>B. 15,001–28,000<br>C. 28,001–55,000<br>D. 55,001–75,000
6.	Where do you live?	A. Italy<br>B. Other country: ______
7.	How many children do you have?	____________
8.	In the course of the study, you will be asked questions about one of your children. It is important that your child is between 2 and 16 years old. If you have more than one child in this age range, we ask you to answer with respect to one of them.<br><br>How old is your child?	____________
9.	Child's sex	A. Female<br>B. Male
10.	Do you have a physical or psychiatric diagnosis?	A. Yes<br>B. No
11.	Are you undergoing psychiatric or psychological treatment?	A. Yes<br>B. No
12.	Does your child have a physical or psychiatric diagnosis?	A. Yes<br>B. No
13.	Is your child undergoing psychiatric or psychological treatment?	A. Yes<br>B. No

Supplementary Material 2. Covid-19 Risk Index questionnaire

1. During this period of COVID-19 emergency, have you continued to work?	A. Yes, regularly, in the same way as before the pandemic<br>B. Yes, regularly, but from home (smart working)<br>C. No, I am not working at the moment (due to holidays, sick leave, etc.)<br>D. No, I lost my job because of the pandemic<br>E. No, I wasn't working before the pandemic either
2. Due to the COVID-19 pandemic, did your child have to attend school lessons from home?	A. Yes<br>B. No
3. If yes, did you have to be responsible for its educational management?	A. Yes<br>B. No
4. If yes, for how many hours per day?	A. 1 – 2 hours<br>B. 2 – 4 hours<br>C. 4 – 6 hours<br>D. More than 6 hours
5. Have you/had you someone to help you in the domestic management of your child/children?	A. Yes<br>B. No
6. How much do you feel the changes due to the COVID-19 pandemic have affected your child's daily management?	A. Not at all<br>B. A little<br>C. Fairly<br>D. Very much
7. Have you had any symptoms related to COVID-19?	A. Yes<br>B. No
8. Have you tested positive for COVID-19?	A. Yes<br>B. No
9. Has any of your family members been affected by COVID-19?	A. Yes<br>B. No
10. If yes, did any of your family members die due to COVID-19?	A. Yes<br>B. No
11. Have any of your friends been affected by COVID-19?	A. Yes<br>B. No
12. If yes, did any of your friends die due to COVID-19?	A. Yes<br>B. No
